# Extending evolutionary forecasts across bacterial species

**DOI:** 10.1098/rspb.2024.2312

**Published:** 2024-12-11

**Authors:** Jennifer T. Pentz, Aparna Biswas, Bassel Alsaed, Peter A. Lind

**Affiliations:** ^1^ Department of Molecular Biology, Umeå University, Umeå, Sweden; ^2^ Los Alamos National Laboratory, Los Alamos, NM, USA; ^3^ Umeå Centre for Microbial Research, Umeå University, Umeå, Sweden

**Keywords:** *Pseudomonas syringae*, *Pseudomonas savastanoi*, experimental evolution, wrinkly spreader, c-di-GMP, evolutionary predictability

## Abstract

Improving evolutionary forecasting requires progressing from studying repeated evolution of a single genotype under identical conditions to formulating broad principles. These principles should enable predictions of how similar species will adapt to similar selective pressures. Evolve-and-resequence experiments with multiple species allow testing forecasts on different biological levels and elucidating the causes for failed predictions. Here, we show that forecasts for adaptation to static culture conditions can be extended to multiple species by testing previous predictions for *Pseudomonas syringae* and *Pseudomonas savastanoi*. In addition to sequence divergence, these species differ in their repertoire of biofilm regulatory genes and structural components. Consistent with predictions, both species repeatedly produced biofilm mutants with a wrinkly spreader phenotype. Predominantly, mutations occurred in the *wsp* operon, with less frequent promoter mutations near uncharacterized diguanylate cyclases. However, mutational patterns differed on the gene level, which was explained by a lack of conservation in relative fitness of mutants between more divergent species. The same mutation was the most frequent for both species suggesting that conserved mutation hotspots can increase parallel evolution. This study shows that evolutionary forecasts can be extended across species, but that differences in the genotype–phenotype–fitness map and mutational biases limit predictability on a detailed molecular level.

## 1. Introduction

The predictability of evolution is a fundamental biological question and the ability to forecast evolutionary events could lead to applications in infectious diseases and cancer, and aid in predicting the ability of organisms to adapt to climate change [1–3]. Traditionally, many biologists have been sceptical of the possibility to predict future evolutionary events given that there are often millions of possible mutations and the dependence of previous events, historical contingencies, is difficult to take into account. Adaptive laboratory evolution replay experiments, where multiple replicates of microbial or viral populations evolve under identical conditions, show that evolution is often surprisingly repeatable, suggesting that it might be possible to predict short-term evolutionary outcomes. However, the results from ‘historical difference experiments’, where replicate lines first evolve separately to evolve different life histories and then evolve in the same environment, show that the effects of historical contingencies can be significant even in cases where the differences are relatively minor (reviewed in [4]). This reduced predictability is commonly explained by epistasis, where the fitness effects of mutations depend on genetic background in closely related strains, and therefore becomes increasing difficult to predict with increasing genetic divergence [5]. Although there are many experimental evolution studies exploring the predictability of evolution in terms of repeatability, it is rarely attempted to forecast experimental evolution in the sense of using ‘data from the past or present to make a prediction about the future’ [6] under significantly different experimental conditions [7].

A related question is whether the evolution of related species can be predicted based on previous data and models from another species. To put it simply, will similar species evolve in similar ways in similar environments? This can be seen as an extreme case of a historical difference experiment in that the historical differences have accumulated over millions of years, including gene loss and gain of genes through horizontal gene transfer. The success of these kinds of evolutionary forecasts will ultimately depend on the conservation of the genotype-to-phenotype map (molecular functions and regulatory interactions) between species, conservation of the relative fitness effects of mutations and conservation of mutational biases (mutational target size and mutational hotspots).

We have previously shown that a mathematical model [8] of the genotype-to-phenotype map constructed for *Pseudomonas fluorescens* SBW25 (hereafter *Pflu*) together with experimental data could be used to predict evolutionary outcomes on various biological levels for the related species *Pseudomonas protegens* Pf−5 (hereafter *Ppro*) [9]. This study demonstrated that the well-established ‘wrinkly spreader’ (WS) model system could be extended to a related species and listed eight predictions of evolutionary outcomes for five more distantly related *Pseudomonas* species. The experimental setup of the WS system is simple: Bacteria are grown under static conditions where strong selection for access to oxygen at the surface leads to increased frequency of mutants with the ability to colonize the oxygen-replete air–liquid interface through adhesion between cells and/or the wall of the growth vessel [10]. Wrinkly spreader mutants can be identified by their divergent colony morphology on agar plates and can be easily isolated even when present at a frequency lower than 1%, with most mutants requiring only a single mutation [9,11,12]. WS mutants all have mutations activating production of c-di-GMP by diguanylate cyclases (DGCs), a conserved signal for biofilm formation resulting in increased production of exopolysaccharides that are the main structural components of the biofilm [7,11,12]. While there are many different DGCs that can be activated by mutation to produce the WS phenotype [12], three mutational pathways dominate in both *Pflu* and *Ppro* (Wsp, Aws and Mws) [8,9,11] that all encode DGCs under negative regulation. These pathways have a larger mutational target size because there are many mutations that can disrupt the negative regulator (e.g. WspF for the Wsp pathway) or its interacting proteins (WspA and WspE) [12]. This leads to a hierarchy of genetic routes to WS where the most commonly used pathways are those under negative regulation [11]. The second most common type is promoter mutations [9,12], a pattern that could be explained by an increased local mutation rate at promoters [13,14]. Although differences in the mutation rate to WS for different genes provide an explanation for why some pathways are more common, fitness differences have also been shown to have a major impact on mutational patterns for the WS system. One example is *wspA,* which has a high mutation rate to the WS phenotype [8], but where mutations are not observed after experimental evolution in *Pflu* or *Ppro* [9,11], which can be explained by their lower competitive fitness [8,9].

Here, we explore if our predictions can be extended to more divergent species with different complements of DGCs and exopolysaccharides. Are more closely related species more likely to evolve in the same way or are evolutionary outcomes more idiosyncratic and dependent on largely unpredictable differences in fitness effects of mutations and mutational hotspots? We test our eight previously published predictions [9] outlined in table 1, for two more distantly related *Pseudomonas* species: *Pseudomonas syringae* pv. tomato DC3000 (hereafter *Psyr*) and *Pseudomonas savastanoi* pv. phaseolicola 1448A (hereafter *Psav*).

**Table 1.** Forecasts for *Psyr* and *Psav* from Pentz *et al.* (2021) [9].

abbrev.	prediction	outcome
P1	mutants with increased ability to colonize the air–liquid interface by increased cell-cell or cell-wall adhesion will evolve and rise to high frequencies	WS mutants found in 32/100 *Psyr* and 37/90 *Psav* populations
P2	mutations in the molecular networks of the negatively regulated DGCs WspR (for *Psyr* and *Psav*), followed by MwsR (for *Psyr* only) will be the most common route to WS due to a large mutational target size	30/32 mutations in Wsp and Mws (figure 1*a*) for *Psyr* and 34/37 mutations in Wsp (figure 1*b*) for *Psav*
P3	a previously described mathematical model [7,12] predicts that most mutations will be in *wspA*, *wspE*, *wspF* for *Psyr* and *Psav*, and *mwsR* for *Psyr* only	*Psyr*: 17 *wspA*, 7 *wspE*, 6 *mwsR*, 0 *wspF* (figure 1*a*)
		*Psav*: 14 *wspA*, 18 *wspE*, 1 *wspC*, 1 *wspR*, 0 *wspF* (figure 1*b*)
P4	mutated regions for proteins in the Wsp and Mws pathways can be predicted based on previous WS mutations in *Pflu* and *Ppro*	yes for WspA and MwsR, no for WspE (electronic supplementary material, figure S2)
P5	the most common type of mutation will be loss of function, followed by mutations in promoter regions then activating and double inactivating mutations	*Psyr*: 29/32 disrupt negative regulation, 3 promoter
		*Psav*: 32/37 disrupt negative regulation, 3 promoter, 2 possibly activating
P6	mutants will have reduced motility due to increased production of c-di-GMP by DGCs, which is a signal for increased production of exopolysaccharides and give the mutants a WS morphotype	*Psyr*: all reduced motility (reconstructed *wspF* small decrease) (figure 2*b*)
		*Psav*: all reduced motility except PSPPH_0905P and *wspF* (figure 2*d*)
P7	mutations will not be evenly spread between the predicted *wspA*, *wspE*, *wspF* and *mwsR* genes with large mutational targets, but found in those where mutations have the largest beneficial fitness effects	*wspF* lower fitness—no mutations found.
		*wspA*, *wspE*, and *mwsR* (for *Psyr*) similar high fitness—commonly found (figure 3)
P8	WS mutants will primarily use exopolysaccharides for increased adhesion as they provide the highest structural stability and fitness with cellulose predicted for *Psyr* and Psl for *Psav*	*Psyr* WS mutants overproduce cellulose (*wss*). *Psav* overproduce alginate (*alg*). But WS mutants with EPS genes deleted have similar fitness as those with EPS genes (figure 4)

Experimental tests of the predictions confirmed that WS mutants with an increased ability to colonize the air–liquid interface evolved for both *Psyr* and *Psav* and that Wsp was the most commonly mutated pathway for both species followed by Mws in *Psyr*. However, the mutational patterns in *Psyr* and *Psav* differed from those found in *Pflu* and *Ppro,* which could partly be explained by fitness differences. This study shows that it is possible to make evolutionary forecasts over larger phylogenetic distances, but that unpredictable differences in mutational hotspots and fitness effects of mutations limit detailed genetic predictions.

## 2. Results

To examine the causes and limitations of evolutionary predictability across longer phylogenetic distances, we used *Psyr* and *Psav* which are further diverged from *Pflu* than *Ppro*, with 75% average amino acid identity in genes commonly targeted by WS mutations compared to 81% for *Ppro* [9] (see electronic supplementary material, table S1 for details). While *Psyr* and *Psav* are more closely related to each other than *Pflu* and *Ppro*, there is still substantial sequence divergence between them (electronic supplementary material, table S1). Our previously published [9] predictions are listed in table 1 and referred to as P1–P8. Predictions were made across multiple biological levels that are presented here in the order that we experimentally evaluated them, which makes the numbering different than in Pentz *et al*. [9]. These predictions are formally independent of each other, but some are closely linked. For example, we would not expect to find mutations in the *wsp* operon unless WS mutants evolve, although this is still possible.

### (a) P1. Mutants with increased ability to colonize the air–liquid interface by increased cell-cell or cell-wall adhesion will evolve and rise to high frequencies

We performed experimental evolution in static growth microcosms for 7 days at 25°C to select for wrinkly spreader (WS) mutants that colonize the air–liquid (AL) interface in both *Psyr* and *Psav*. Each replicate population was started from a single colony with wild-type morphology ensuring that any mutants with WS colony morphology evolved independently. Colonization of the AL interface was observed and 32 WS mutants were isolated for *Psyr* and 37 mutants for *Psav,* each mutant from a different population.

### (b) P2. Mutations in the molecular networks of the negatively regulated DGCs WspR (for Psyr and Psav), followed by MwsR (for Psyr only) will be the most common route to WS due to a large mutational target size

**Figure 1.**
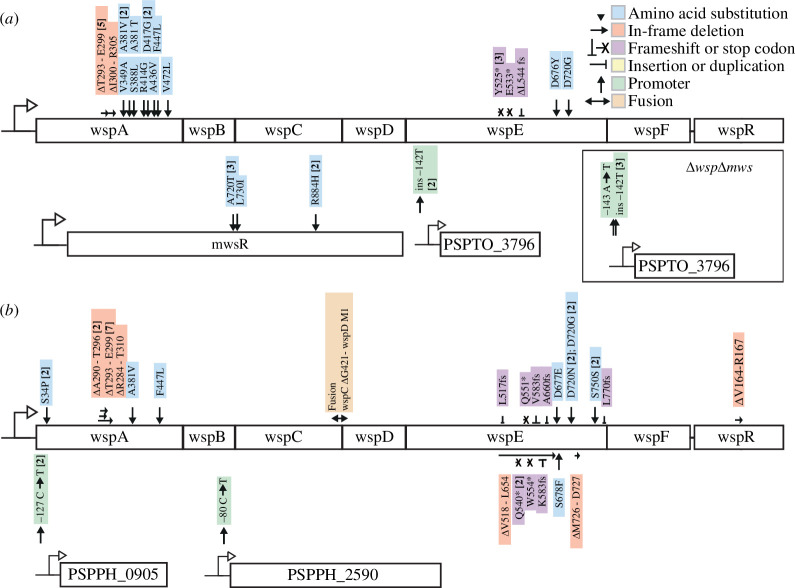
Mutations identified in WS mutants. (*a*). Thirty-two independent WS mutants were isolated for *Psyr*. Four additional WS mutants were identified after experimental evolution with Δ*wsp* Δ*mws* double deletion mutant. (*b*). Thirty-seven independent mutants were isolated for *Psav* after experimental evolution. Numbers in brackets are the number of independent mutants found. Details are available in electronic supplementary material, table S2.

To further evaluate our predictions, we determined the genetic cause of WS evolution for all isolated mutants using a combination of Sanger and Illumina sequencing. First, we examined the most common pathways targeted by mutations to determine if these were primarily found in the predicted DGC pathways. Compared to *Pflu*, *Psyr* and *Psav* have a different repertoire of available DGCs [9]. Importantly, they do not encode all three common genetic pathways (Wsp, Aws, MwsR) to WS used in *Ppro* [9] and *Pflu* [11] where they account for approximately 99% of WS mutants [8]. *Psyr* encodes Wsp and MwsR (but not Aws), while *Psav* only encodes Wsp. Despite these major differences, most mutations were found in the predicted common pathways available, with 30/32 mutations occurring in Wsp and Mws (figure 1*a*) for *Psyr* and 34/37 mutations occurring in Wsp (figure 1*b*) for *Psav*.

**Figure 2.**
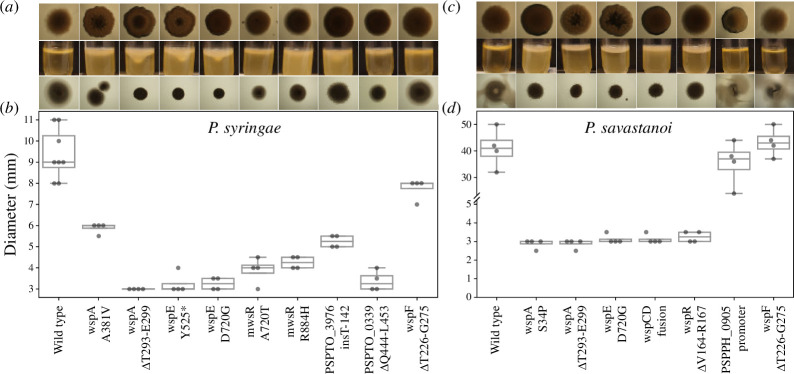
Phenotypic characterization of reconstructed mutants. (*a*) *Psyr* colony morphology (1.5% agar), biofilm formation under static growth and swimming motility (0.3% agar) of reconstructed mutants. (*b*) Swimming motility of *Psyr* reconstructed mutants. (*c*) *Psav* colony morphology (1.5% agar), biofilm formation under static growth and motility (0.3% agar) of reconstructed mutants. (*d*) Swimming motility of *Psav* reconstructed mutants.

**Figure 3.**
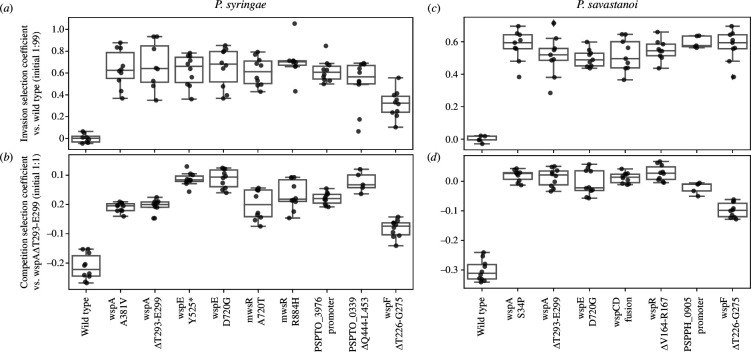
Fitness of reconstructed *Psyr* and *Psav* WS mutants. Invasion fitness (*a,c*) of mutants was measured against the wild-type (initial ratio 1:99 mutant: wild-type), while competition fitness (*b,d*) was measured against the most common WspA mutant (initial ratio 50:50). Reciprocal pairwise competitions were performed in YFP and BFP backgrounds.

### (c) P3. Most mutations will be in *wspA*, *wspE*, *wspF* for *Psyr* and *Psav* and *mwsR* for *Psyr* only

A previously described mathematical model [8,15] predicted that most mutations would be in *wspA*, *wspE*, and *wspF* for *Psyr* and *Psav* and *mwsR* for *Psyr*. This prediction is based on the assumption that disabling mutations, decreasing reaction rates, are more probable than enabling mutations. The majority of the mutations were found in the predicted genes (*Psyr* 30/32, figure 1*a*, and *Psav* 32/37 mutants, figure 1*b*). However, mutational patterns at the gene level were strikingly different when compared with the previously isolated *Pflu* and *Ppro* WS mutants (electronic supplementary material, figure S1). The most obvious difference was the lack of mutations in *wspF*, which is surprising as it encodes the negative regulator of the Wsp network and any inactivating mutation is expected to result in a WS phenotype. Another marked difference is that there are many mutations in *wspA* (16 for *Psyr* and 14 for *Psav*), which has a high predicted mutation rate to WS [8], but where mutations were not found after experimental evolution for *Pflu* and *Ppro* [9,11]. The mathematical model also predicted low rates of *wspC* and *wspR* WS mutations because they are expected to require enabling (gain-of-function) mutations and for *Psav* these genes were found to be mutated once each (figure 1*b*) while no mutations were found there for *Psyr*.

### (d) P4. Mutated regions for proteins in the Wsp and Mws pathways can be predicted based on previous WS mutations in *Pflu* and *Ppro*

In our previous work, we reported that mutational patterns in *Pflu* proteins could be used to successfully predict mutational regions in *Ppro* [9], suggesting these predictions could be extended to *Psyr* and *Psav*. Here, we find that mutated regions were successfully predicted for WspA and MwsR (electronic supplementary material, figure S2), but there was a clear difference in mutational patterns for the types of mutations observed in WspE. Mutations in WspE have previously been found in both *Pflu* [8,11] and *Ppro* [9], but they are in all cases missense mutations near the phosphorylation site (4-aspartyl phosphate at D720) in the response regulatory domain. These mutations were predicted [9] to lead to loss-of-function of the phosphorylation reaction that activates WspF leading to disabling of negative regulation and constitutive activation of WspR. For both *Psyr* and *Psav*, missense mutations in the same region are also found, but there were many different insertion and deletion mutations, including frameshifts and nonsense mutations causing loss of the entire CheW-like and response regulatory domains (electronic supplementary material, figure S2). Thus, mutated regions could not be predicted based on previous data from *Pflu* and *Ppro* for the WspE protein (electronic supplementary material, figure S2).

### (e) P5. The most common type of mutation will be loss-of-function, followed by mutations in promoter regions then activating and double inactivating mutations

As described in the previous section, WS mutations in WspE commonly result in loss-of-function of the CheW-like and response regulatory domains. The most common mutation found for both *Psyr* and *Psav* is a WspA T293-E299 deletion that is predicted to lead to loss-of-function of the methylation sites in the region 280–310 [9]. Both WspE and WspF mutations are thus likely to disable the functional interaction with the negative regulator WspF.

In *Psyr*, two mutations were found upstream of PSPTO_3796 (figure 1*a*), encoding a putative DGC predicted to be localized to the cytoplasmic membrane. To determine if there are also other DGCs that can be activated by rare mutations, as previously demonstrated in *Pflu* [12], *wspABCDEFR* and *mwsR* were deleted in *Psyr*, followed by experimental evolution using an identical protocol as for the ancestor. This allowed isolation of four additional independent WS mutants that all had mutations in the PSPTO_3796 promoter (figure 1*a*). Similarly, in *Psav*, two mutations occurred in the promoter region upstream of PSPPH_0905 and one mutation upstream of PSPPH_2590 (figure 1*b*), both encoding proteins with DGC domains.

### (f) P6. Mutants will have reduced motility due to increased production of c-di-GMP by DGCs

All WS mutants isolated for both *Psyr* and *Psav* have mutations linked to DGCs, presumably increasing production of c-di-GMP (figure 1*a*,*b*). To assess our phenotypic predictions and further examine the causes of the observed mutation patterns, we reconstructed a subset of mutants representing the diversity of mutations for *Psyr* and *Psav*, creating two independent identical mutants in ancestral strains expressing either of two fluorescent proteins (YFP and BFP). This ensures that the identified mutation is the sole cause of the phenotypic changes observed and that there are no secondary mutations influencing the results. We also constructed strains with mutations in *wspF* and PSPTO_0339 (*dgcH*) that were predicted to be observed at high frequency [9] based on previous data [12] but were not found after experimental evolution.

Swimming motility was clearly reduced for most mutants (figure 2*b*,*d*), but not for *wspF* for both *Psyr*/*Psav* or for PSPPH_0905. Unlike the other reconstructed mutants, the *wspF* and PSPPH_0905 mutants did not form thick biofilms (figure 2*a*,*c*). Furthermore, the colony morphology for the reconstructed WS mutants was most pronounced for *wspA* and *wspE* mutants, but the wrinkly phenotype was less characteristic than for WS mutants in *Pflu* and *Ppro* (figure 2*a*,*c*).

### (g) P7. Mutations will not be evenly spread between the predicted *wspA*, *wspE*, *wspF* and *mwsR* genes with large mutational targets, but found in those where mutations have the largest beneficial fitness effects

To determine how differences in fitness between WS mutants could explain the observed mutational patterns, we conducted two types of competitive fitness assays, similar to previous studies [8,9,12,16]. The first assay, measuring what we refer to as ‘invasion fitness’, measures competition of a WS mutant starting at 1% against the wild-type ancestor at 99% and is intended to measure fitness at the early stages of AL interface colonization. In the other assay, measuring ‘competition fitness’, each WS mutant is competed 1:1 against a *wspA* reference WS mutant that represents the most common mutant isolated after experimental evolution for both *Psyr* and *Psav*. These assays can provide data to explain the absence of expected mutants, like *wspF* loss-of-function mutants, after experimental evolution. They can also determine if rare promoter mutants and mutants with potential activating mutations have similar fitness to the commonly mutated pathways, which would support the hypothesis that mutational target size is a main determinant of evolutionary outcomes in the WS model system [12].

All WS mutants had similar invasion fitness against the ancestor and the reference strain (WspA ΔT293-E299) except for *Psyr* WspF that is, substantially lower, but still significantly positive (*p* = 0.002; one-way ANOVA, *F_9,86_* = 16.7, *p* < 0.0001, pairwise differences assessed with Tukey HSD) (figure 3*a*,*c*). Thus, all reconstructed WS mutants are expected to be able to rapidly increase in frequency when appearing in an ancestral population.

The competition assay showed that in *Psyr,* both WspE mutants had higher fitness than the WspA reference (figure 3*b*; WspE Y525* *p *< 0.0001, WspE D720G *p *< 0.0001; one-way ANOVA, F_9,85 _= 68.5, *p *< 0.0001, pairwise difference assessed with Tukey HSD). Surprisingly, the PSPTO_0339 mutant also had higher fitness (figure 3*b*; *p *< 0.0001) despite it not being observed after experimental evolution. The other *Psyr* WS mutants had similar fitness as the WspA reference except for the WspF mutant, which could explain why WspF mutants were not observed after experimental evolution (figure 3*b*). The WspF mutant also had clearly reduced competition fitness for *Psav* (figure 3*d*) while the other WS mutants had similar fitness as the WspA reference, with the PSPPH_0905 mutant showing a small but insignificant decrease (WspF *p *< 0.0001, one-way ANOVA, F_7,67 _= 149.1, pairwise differences assessed with Tukey HSD). The difference in the ratio of *wspF* to other WS mutants between *Pflu*/*Ppro* and *Psyr*/*Psav* is highly unlikely to be due to insufficient sampling (*p* = 10^−10^, Fisher’s exact test), strongly suggesting that fitness differences are the main factor explaining the lack of WspF mutants for *Psyr* and *Psav* after experimental evolution.

### (h) P8. WS mutants will primarily use exopolysaccharides for increased cell-cell adhesion, with cellulose predicted for *Psyr* and Psl for *Psav*

To test the prediction that mutants with increased ability to colonize the AL interface would use exopolysaccharides as a key adhesive structural component, we used Tn5 transposon mutagenesis of WS mutants to isolate insertion mutants that reverted to the ancestral colony morphology. In *Psyr*, we found insertions in *wssA*, *wssB*, *wssC*, *wssE*, *wssF* and *wssI,* which encode proteins for cellulose biosynthesis, suggesting that the divergent colony morphology is caused by increased production of cellulose. This aligned with previous predictions [8] that cellulose will be the main EPS structural component utilized to colonize the air–liquid interface when available. *Psav*, on the other hand, does not encode genes for biosynthesis of cellulose and Psl was predicted [9] to be used as the main structural component. We did a similar Tn5 mutagenesis screen for *Psav* in WspA mutants and found insertions in the alginate operon genes *algI*, *algX*, *alg44* and *alg8*, which instead suggests that increased alginate production is the main cause of the divergent colony morphology.

**Figure 4.**
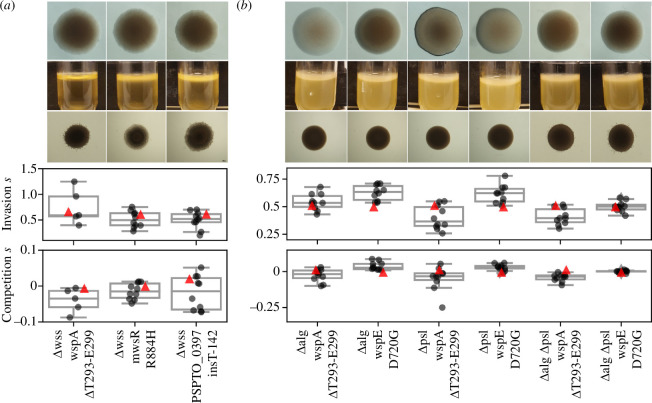
Deletion of known exopolysaccharides biosynthesis genes does not decrease fitness. (*a*) *Psyr* WS mutants with deletion of the *wss* operon. From top: colony morphology (1.5% agar), biofilm formation under static growth and swimming motility (0.3% agar) of reconstructed mutants. Invasion and competition fitness of identical WS mutants with the *wss* operon intact shown as red triangles for comparison (from figure 3). (*b*) *Psav* WS mutants with deletion of the *alg* and/or *psl* operons. Colony morphology (1.5% agar), biofilm formation under static growth, and swimming motility (0.3% agar) of reconstructed mutants. Invasion and competition fitness of identical WS mutants with the *alg* and *psl* operons intact shown as red triangles for comparison (from figure 3).

While the Tn5 mutagenesis experiments indicated that increased production of cellulose in *Psyr* and alginate for *Psav* cause wrinkly colony morphology, it is not necessarily the main structural component required for successful AL interface colonization leading to increased fitness. Therefore, we deleted the *wss* operon from several WS mutants of *Psyr* and the *alg* operon from WS mutants of *Psav* to determine the roles of these exopolysaccharides in biofilm formation under static conditions. Surprisingly, neither of these deletions reduced fitness in the invasion assay or the competition assay (figure 4*a*). We also constructed strains of WS mutants in *Psav* with the *psl* operon deleted or both *alg* and *psl* operons deleted, but no reduction in either invasion or competition fitness was observed (figure 4*b*). These results suggest that these exopolysaccharides, although apparently increasingly produced in WS mutants, do not contribute significantly to increased fitness or that, in their absence, other unknown adhesive factors can substitute for their function.

### (i) Proteinase K delays biofilm formation in *Psyr* WS mutants lacking cellulose biosynthesis genes

To further investigate the molecular basis of biofilm formation in WS mutants devoid of cellulose production for *Psyr* and Psl and alginate production for *Psav* we used proteinase K that cleaves peptide bonds, cellulase that cleaves β−1–4 glycosidic bonds present in some polysaccharides or DNAse that cleaves phosphodiester linkages in DNA. After 48 h of incubation with proteinase K a reduction in biofilm formation at the air–liquid interface was apparent for cultures of *Psyr* Δ*wss* WspA ΔT293-E299 and to a lesser degree for *Psyr* Δ*wss* MwsR R884H, which could indicate that an adhesive protein or peptide plays a major structural role (electronic supplementary material, figure S3). However, addition of proteinase K only delayed biofilm formation and at 72 h it was clearly visible at the AL interface for both mutants (electronic supplementary material, figure S3). None of the three enzymes visibly reduced biofilm formation in *Psav* Δ*alg* Δ*psl* WspA ΔT293-E299 (electronic supplementary material, figure S3).

## 3. Discussion

Explicit testing of short-term evolutionary forecasts allows an unbiased view of the factors that govern evolutionary processes and critical examination of fundamental biological assumptions. Here, we show that short-term evolutionary forecasts are to some degree extendable to similar species evolving in similar environments, but that differences between species can make evolutionary outcomes idiosyncratic and unpredictable on the molecular level. Several of our previously published predictions [9] were successful (table 1), including the evolution of mutants with increased ability to colonize the AL interface (P1) and the mutational activation of c-di-GMP production resulting in reduced motility (P6). Interestingly, the mutants with higher motility and lower biofilm formation (figure 2) have lower fitness (figure 3) in competitions. This suggests a link between reduced motility, increased biofilm formation and increased fitness that may be related to the amount of c-di-GMP produced by the mutationally activated DGCs. The mutational pattern of common loss-of-function mutations, resulting in loss of a key function of the protein, and more rare promoter mutations (P5) was observed in all four species tested so far [9,12], suggesting that the mutational target size is a major determinant of evolutionary outcomes in this model system where multiple genetic pathways to similar high-fitness phenotypes are available. This is also reflected in the conservation of the order of frequency of pathways used (P2) for all four species tested (this work and [9,11]). The same pattern has also been observed in a similar experiment with *Pseudomonas simiae* PICF7 [17].

At the gene level, there is less overlap with the previous studies in *Pflu*/*Ppro*, where mutations in the *wsp* operon were found primarily in *wspF*, while for *Psyr*/*Psav* mutations, no mutations were found in *wspF* and most mutations were in *wspA* and *wspE*. This aligns well with P7 that mutations will be unevenly spread between the genes predicted by a previously developed model of the molecular networks [8] and clustered in the genes where mutations can provide the largest beneficial fitness effects. It was confirmed that fitness effects provide a reasonable explanation for these patterns in all four species ([8,9] and this work) and it might also explain observations in the even more distantly related *Burkholderia cenocepacia* where only mutations in *wspA* and *wspE* were found [18]. Another example of differences in fitness effects is WS mutations in WspR that are expected to be relatively frequent [8,19] but that are not found after experimental evolution in *Pflu* as they have much lower fitness [8], while in *Psav* the WspR mutant had similar fitness as other WS mutants (figure 3*c*,*d*). Clearly an inability to predict which genes will harbor mutations that lead to the most fit mutants will limit the success of evolutionary forecasts.

The relative fitness of WS mutants is not necessarily the only reason for the differences in mutational patterns between species. The difference in the mutation spectrum in WspE hints that its molecular function or interactions are in some way different. *Pflu*/*Ppro* WspE only had very specific point mutations around an active site, while *Psyr*/*Psav* also had mutations causing loss of the entire CheW-like and response regulatory domain by a range of different indels and point mutations. This could bias the observed mutational patterns towards WspE due to an increased mutational target size. Thus, the mutated regions and types of mutations for WspE could not be predicted based on previously observed mutations in other species (P4; electronic supplementary material, figure S2). In contrast, there was a strong similarity between mutations in WspA in *Pflu* [8] and in *Psyr*/*Psav,* even if exact molecular parallelism was rare (electronic supplementary material, figure S1).

The most obvious forecasting failure is the limited role played by the exopolysaccharides cellulose for *Psyr* and alginate and Psl for *Psav* (P8). Although it seems likely that the exopolysaccharides are indeed produced at a higher level in WS mutants, based on the difference in colony morphology of deletion mutants, there was no reduction in fitness either in invasion or competition assays. The ability of proteinase K to delay biofilm formation in *Psyr* WS mutants with the cellulose biosynthesis operon deleted suggests that a protein or peptide plays a role in increasing the ability to colonize the AL interface. However, after extended incubation there is biofilm formation exceeding that of the ancestor indicating that adhesive factors under c-di-GMP control resistant to degradation by proteinase K are still produced.

By repeatedly conducting experimental tests of forecasts of WS evolution for different species there are opportunities to use new data to iteratively improve forecasts. The mathematical model used in [8] that formed the basis for the predictions [9] on the gene and pathway levels only included the regulatory connections of the main DGC networks. An alternative approach is to use the mutational patterns accumulated by experiments in different species (electronic supplementary material, figure S1) to estimate the mutational target size to the WS phenotype for each gene. The success of such a model is expected to be limited by the presence of mutational hotspots. In *Pflu*, a reporter construct was used to show that just two mutations account for 42% of the total mutation rate to WS [8]. Another hotspot in the *nlpD* gene in *Pflu* was recently shown to have a mutation rate 5000-fold higher than expected from genomic average mutation rate resulting in an extreme case of parallel molecular evolution [13]. Although it is not generally feasible to directly measure the impact of mutation hotspots in WS experimental evolution, as this would require separating the effects of mutation and selection, repeated mutations are common. A single mutation accounts for 26% of WS mutants observed in *Ppro* [9] and in this study we find that the same mutation in *wspA* accounts for 16% of mutants in both *Psyr* and *Psav*. These mutants were found to have similar fitness as less frequent mutants suggesting that an increased mutation rate is the main cause of their high frequency.

Differences in mutation hotspots between species can bias evolutionary outcomes, but it is not obvious to what extent this will limit predictability. This problem was addressed by Sun and Lind in [20] by combining a target size model of the WS model system with a distribution of mutation rates to examine how much variation in mutation rates would affect evolutionary outcomes on the levels of individual mutations, genes, and pathways. Based on repeated observations that mutational hotspots can have major effects on evolutionary outcomes [8,9,16,21–25], it is clear that a distribution of mutation rates must have a long-tail with a small fraction of mutations that have greatly increased mutation rates [20]. The theoretical predictions from [20] align well with the experimental data for the different *Pseudomonas* species ([8,9,11] in that it shows that even with the inclusion of strong mutation hotspots, evolution on the pathway level is expected to be predictable between species (P2) so that Wsp is typically the most commonly mutated pathway.

Another aspect that can also be explained by the distribution of mutation rates model [20] is why the rare pathways used for the different species are likely to be different. Most DGCs are not under negative regulation and they must be activated by mutations in promoter regions or specific intragenic activating mutations to produce WS mutants [12]. Given that there are many putative DGCs for each species (39 in *Pflu*, 39 in *Ppro*, 35 in *Psyr* and 34 in *Psav*), a distribution of mutation rates can make evolution idiosyncratic for pathways with small mutational target sizes [20]. Even though at least nine different DGCs in *Pflu* can be activated by promoter mutations or specific intragenic mutations [12] and seven of these are present in both *Psyr* and *Psav* [9] they were not found to be mutated here. Thus, although the general prediction that promoter mutations will be the second most common type of mutations (P5) is often successful, it is not possible to predict to which DGCs those promoters will belong to, as this is likely determined by the mutation rates of a few nucleotides in the promoter region of each DGC.

Our results show that short-term evolutionary forecasting in simple environments is possible at higher biological levels even for species that are estimated to have diverged millions of years ago [26] and that have gained and lost significant parts of their genomes. We find that at the lower biological levels of individual genes and mutations evolution is less predictable but still highly repeatable within a single strain. By demonstrating that the well-established wrinkly spreader model system can be extended to even more divergent *Pseudomonas* species we see great promise in continuing to develop this model system that could allow direct testing of evolutionary forecasts across the hundreds of known diverse *Pseudomonas* species [27]. While many of the most basic predictions could turn out to be generally applicable, it will be of particular interest to also see the exceptions to the rules, especially if the underlying causes can be elucidated instead of simply referring to undefined historical contingencies or epistasis. These forecasting failures can provide increased understanding of the causes underlying the predictability of evolutionary processes, especially when combined with construction of expected mutants not observed after experimental evolution. These insights can then be used to inform and critically evaluate machine-learning models for evolutionary forecasting that can easily incorporate factors like gene content and phylogenetic relationships but where mutational hotspots can introduce biases in training data and a few nucleotide sequence differences can drastically alter evolutionary outcomes [13,21].

## 4. Material and methods

### (a) Strains and media

We used *Pseudomonas syringae* pv. tomato DC3000 (*Psyr*, GenBank: genome AE016853.1, plasmids AE016854.1 and AE016855.1 [28] and *Pseudomonas savastanoi* pv. phaseolicola 1448A (*Psav*, Genbank genome CP000058.1, plasmids CP000059.1 and CP000060.1, [29]) and its derivatives for all experimental evolution and phenotypic characterization. Cloning of PCR fragments was performed using *E. coli* DH5α *λpir. Psyr* and *Psav* were cultured in tryptic soy broth (TSB) supplemented with 10 mM MgSO4 and 0.2% glycerol (TSBGM) for experimental evolution and fitness assays. Genetic engineering was performed using Lysogeny broth (LB), while counter-selection of the *sacB* marker was performed using LB without NaCl supplemented with 8% sucrose. Solid media consisted of 1.5% agar added to LB or TSBGM with or without 10 mg l^−1^ Congo red. Motility assays were conducted in 0.3% agar TSBGM. We used gentamicin (10 mg l^−1^) and kanamycin (50 mg l^−1^) for *E. coli*, *Psyr* and *Psav,* and nitrofurantoin (50 mg l^−1^) was used to inhibit growth of *E. coli* donor and helper cells after conjugation. All strains were stored at −80°C in LB with 10% DMSO.

### (b) Experimental evolution

For experimental evolution, 1 mL of TSBGM was inoculated with about 10^3^ cells from overnight cultures and grown in a deep well plate (1.1 ml, round walls, polypropylene, Axygen Corning Life Sciences) at 25°C for seven days, without shaking. These overnight cultures were started from different single colonies all exhibiting a smooth wild-type phenotype to ensure that observed WS mutants were not due to standing genetic variation in the founding clone’s freezer stocks ensuring that all WS mutants evolved independently. To reduce possible edge effects due to increased evaporation, the wells at the plate’s edges were left unused and served as contamination controls. To sample cells, a 1 µl plastic loop was utilized to collect cells from the bottom, edges, and surface of the air–liquid interface, which were then transferred to an Eppendorf tube containing LB, and vortexed vigorously. In total 100 replicate populations of *Psyr* and 90 replicate populations of *Psav* were sampled to collect a minimum of thirty WS mutants for each species. After 7 days of incubation, suitable dilutions were plated on TSBGM plates supplemented with Congo red and incubated for another 48 h at 25°C. To identify colonies with WS morphology, the plates were screened, and one colony per well was randomly selected based on its position on the agar plate. A total of 32 independent WS mutants for *Psyr* and 39 WS mutants for *Psav* were obtained. Note that this sampling method does not necessarily reflect the frequency of mutants for the entire population and lack of observed WS mutants on the agar plate does not mean that they are not present in the population at a lower frequency than about 1%. The WS mutants were streaked for single cells twice before overnight growth in LB and freezing. The same protocol was used for the *Psyr Δwsp Δmws* strain where four additional mutants were isolated from 30 replicate populations.

### (c) Genetic engineering

Seven *Psyr* WS mutations and six *Psav* WS mutations representing all mutated genes were reconstructed in the wild-type strains constitutively expressing either a YFP or BFP marker for use in fitness assays and to confirm the role of the mutation in causing the adaptive phenotype. We also introduced a *wspF* ΔT226-G275 mutation previously found to cause a high fitness WS phenotype in *Pseudomonas fluorescens* SBW25 [8] in *Psyr* and *Psav* fluorescent strains and a PSPTO_0339 ΔQ444-L453 mutation previously found in *Ppro* (corresponding to Q493-L503) [9]. This was accomplished by introducing the mutations or deletion constructs into the wild-type *Psyr* or *Psav* using a two-step allelic replacement protocol. The protocol involved the utilization of the mobilizable pEX18Gm suicide plasmid (GenBank AF047518.1) and was carried out as previously described [9] except at 25°C and without heat shock of recipient strains. Deletion of PSPTO_1026-PSPTO_1034 (Δ*wssABCDEFGHI*) in *Psyr* and deletion of PSPPH_1107–1118 (Δ*algABCDEFGHIJKLX*) and PSPPH_3222–3232 (Δ*pslABCDEFGHIJK*) in *Psav* were also done using the same method. DNA fragments for the mutation of *wspF* in *Psyr* and *Psav* and for deletion of mwsR (PSPTO_4631) in *Psyr* were made by gene synthesis (Thermo Fischer Scientific, GeneArt). DNA fragments for deletion of the *wspABCDEFR and wssABCDEFGHI* in *Psyr* and deletion of *algABCDEFGHIJKLX* and *pslABCDEFGHIJK* in *Psav* were prepared using SOE-PCR to generate fragments surrounding the operons as previously described [9,16].

Strains of *Psyr* and *Psav* expressing fluorescent proteins were constructed using a mini-Tn7 transposon that allows site-specific integration downstream of the *glmS* gene. pUC18R6K-mini-Tn7T-Gm, expressing sYFP2 (KM018300.1) or mTagBFP2 (KM018299.1) from a J23101 promoter (https://parts.igem.org/Part:BBa_J23101) and a BBa_B0034 ribosomal binding site (https://parts.igem.org/Part:BBa_B0034) was inserted between the ApaI and KpnI (mTagBFP2) or PstI and KpnI sites (sYFP2). The pUC18R6K-mini-Tn7T-Gm plasmid [30] was a gift from Herbert Schweizer (Addgene plasmid # 65022 ; https://n2t.net/addgene:65022 ; RRID:Addgene_65022) The pFLP3 plasmid (Addgene #64946, [31]) was then introduced by conjugation to remove the FRT-flanked gentamicin resistance marker by FLP recombination. All strain constructions were confirmed with Sanger sequencing of the genomic region modified. All oligonucleotide primers are available in electronic supplementary material, table S3.

### (d) Fitness assays

Two types of competition fitness assays were performed following previously established protocols [9,12,16], except that flow cytometry was used to estimate the frequency of BFP versus YFP cells in a population. We used a BioRad ZE5 cell analyzer flow cytometer to count 100 000 cells for each fitness assay replicate time point apart from *Psav* time 0 points, where we counted 200 000 cells and gated on SSC-H and SSC-W to focus on single cells. We utilized the small particle optical detector, which measures forward scatter with a different laser that can resolve particles as small as 0.3 µm in diameter. A 488 nm laser combined with 530/30 filter was used for counting sYFP2 positive cells and the 405 nm laser combined with a 450/50 filter was used to quantify mTagBFP2 cells.

The first fitness assay measures the ability of a WS mutant to increase from the low frequency in a dominant ancestral population. We intend this setup to be similar to the earliest stage of AL interface colonization where a rare WS mutant attaches and grows at the surface with limited competition from other mutants. This is referred to here as ‘invasion fitness’ and the assay was conducted by mixing the WS mutant with the wild type at a 1:99 starting ratio using a 1000-fold dilution of the wild type from an overnight culture. The assay was conducted under the same conditions as for the experimental evolution for 48 h.

In the second assay, we instead focus on ‘competition fitness’ where a WS mutant competes 1:1 with a WS reference strain over 24 hours under the same conditions as for the experimental evolution starting with a 1000-fold dilution of overnight cultures. The reference strains have a WspA T293-T299 mutation in both *Psyr* and *Psav* and were chosen because it was the most common mutation for both *Psyr* and *Psav*. We calculated selection coefficients (s) as previously described [32] s = [ln(R(t)/R(0))]/[t] where R is the ratio of the mutant to the reference and t is the number of generations of the entire population during the experiment. For both assays, a value of s = 0 represents equal fitness, positive values increased fitness and negative values mean decreased fitness relative to the reference strain. We quantified the fitness cost of the sYFP2 marker compared to mTagBFP2 marker in both the ancestral strains and the WspA reference strains and adjusted the selection coefficient based on this (*Psav* WT =+0.017, *Psav* WspA =+0.019, *Psyr* WT = −0.027, *Psyr* WspA =+0.024). Source data for all figures 3 and 4 are available in electronic supplementary material, table S4.

### (e) Motility assays

Motility assays were conducted to measure the swimming ability of the mutants compared to the ancestor. The assays were performed in TSBGM plates containing 0.3% agar (BD), and the diameter of the strains was measured after 24 h of growth at room temperature. Each strain was tested in duplicates on two separate plates, resulting in four replicates.

### (f) Enzymatic assay

Three hydrolytic enzymes, cellulase (Tokyo Chemical Industries Co., Tokyo (Japan)), proteinase K (Thermo Scientific) and DNAse I (Thermo Scientific) were used to probe the structural basis of biofilm formation at the air–liquid interface. Clones derived from −80°C were streaked onto LB agar plates to obtain a single colony, which was inoculated into 2 ml TSBGM and incubated at 25°C shaking overnight. Overnight cultures were diluted 1000-fold in 2 ml TSBGM with either cellulase (approx. 425 U (25  mg)), proteinase K (approx. 12 U (400  µg)), DNAse I (approx. 10 U (10  µl)) or no hydrolytic enzyme and incubated statically at 25°C. Biofilm formation was photographed at 24 h, 48 h and 72 h.

### (g) Tn5 transposon mutagenesis

We used transposon mutagenesis of WS mutants to identify candidate genes required for the WS colony morphology [33] including putative structural components required for colonization of the air–liquid interface. The pCM639 plasmid containing the IS-Ω-kan/hah transposon was transferred by conjugation from *E. coli* SM10 *λpir* into WS mutants using helper plasmid pRK2013. We selected transconjugants on TSBGM plates supplemented with Congo Red, kanamycin and nitrofurantoin, for counter-selection of *E. coli*. Fewer than 1000 colonies from each independent conjugation were screened for loss of the WS colony morphology and reversion to an ancestral one. The transposons' chromosomal insertion points were then determined by arbitrarily-primed PCR and Sanger sequencing [34].

### (h) DNA sequencing

We sequenced the genomes of 11 WS mutants for *Psav* and identified mutations in *wspA* (PSPPH_3881), *wspE* (PSPPH_3877), *wspR* (PSPPH_3875), *wspC* (PSPPH_3879), PSPPH_0905 and PSPPH_2590. We also sequenced the genomes of four *Psyr* WS mutants that had mutations in *wspA* (PSPTO_1493), *wspE* (PSPTO_1497), PSPTO_3796 and *mwsR* (PSPTO_4631). Sanger sequencing of these candidate genes was then used to find the mutations causing the WS phenotype in the other mutants (electronic supplementary material, table S2).

Genomic DNA was isolated with Genomic DNA Purification Kit (Thermo Fisher) for genome sequencing. For *Psyr*, sequencing libraries were prepared from 1 μg DNA using the TruSeq PCR-free DNA sample preparation kit (cat# FC- 121−3001/3002, Illumina Inc.). The library preparation was performed according to the manufacturers’ instructions (guide#15036187). Sequencing was performed with MiSeq (Illumina Inc.) paired-end 300 bp read length and v3 sequencing chemistry. Sequencing was performed by the SNP&SEQ Technology Platform in Uppsala. The facility is part of the National Genomics Infrastructure (NGI) Sweden and Science for Life Laboratory. The SNP&SEQ Platform is also supported by the Swedish Research Council and the Knut and Alice Wallenberg Foundation. For *Psav* we used a standard miniaturized protocol to prepare DNA libraries for sequencing using the NEBNext Ultra II FS DNA Library Prep Kit for Illumina (New England BioLabs Inc. (Ipswich, MA, USA)). The DNA libraries were quantified using a Qubit dsDNA HS kit and a Qubit 2.0 Fluorometer (Thermo Fisher Scientific Inc. (Waltham, MA, USA)). We used a MiSeq system (Illumina Inc. San Diego, CA, USA) to perform 250 bp paired end sequencing on the prepared libraries (Illumina MiSeq Kit V2 500 cycles).

Sequencing data were analyzed using Geneious Prime software for Mac (v2022.0.2) with reads assembled against the *Psyr* (GenBank: genome AE016853.1, plasmids AE016854.1 and AE016855.1 [28] or *Psav* genome sequences (Genbank genome CP000058.1, plasmids CP000059.1 and CP000060.1, [29]). Complete sequencing data for these clones is available under NCBI BioProject PRJNA1075102. Sanger sequencing was performed by Eurofins Genomics and used to sequence candidate genes to find adaptive mutations and to confirm reconstructed mutations. Primer sequences are available in electronic supplementary material, table S3.

## Data Availability

Raw sequencing reads are available from NCBI Sequence Read Archive (SRA) under BioProject PRJNA1075102. Supplementary material is available online at [35].
